# Role of e-health in addressing sarcopenic obesity: a scoping review protocol

**DOI:** 10.1136/bmjopen-2025-103773

**Published:** 2025-11-04

**Authors:** Mette Nørtoft, Signe Graungaard, Gideon Onyedikachi Iheme, Vasiliki Karagianni, Rebeka Bereczky, Lars Ellegaard, Anne-Marie Boström

**Affiliations:** 1 Hammel Neurorehabilitation and Research Clinic, Department of Clinical Medicine, Aarhus University, Aarhus, Denmark; 2 Department of Nursing, VIA University College, Aarhus, Denmark; 3 Aalborg Universitetshospital, Aalborg, Denmark; 4 Department of Food Studies, Nutrition and Dietetics, Uppsala University, Uppsala, Sweden; 5 Department of Human Nutrition and Dietetics, Michael Okpara University of Agriculture, Umudike, Nigeria; 6 Blodtrycksdoktorn AB, Bredbandet 1, Kalmar, Sweden; 7 Yazen Health AB Spolegatan 22, Lund, Sweden; 8 Institute of Medicine, Sahlgrenska University Hospital, Gothenburg, Sweden; 9 Department of Internal Medicine and Clinical Nutrition, University of Gothenburg, Gothenburg, Sweden; 10 Department of Neurobiology, Care Sciences and Society, Karolinska Institutet, Huddinge, Sweden; 11 Theme Inflammation and Aging, Karolinska University Hospital, Huddinge, Sweden; 12 R&D Unit, Stockholms Sjukhem, Stockholm, Sweden

**Keywords:** Obesity, eHealth, GERIATRIC MEDICINE

## Abstract

**Abstract:**

**Introduction:**

The global burden of malnutrition is compounded by the challenges of obesity and sarcopenia, a combination known as sarcopenic obesity. This condition, defined by increased fat mass alongside declining muscle mass and function, poses significant health risks, including metabolic dysregulation and cardiovascular complications. Despite its growing prevalence and clinical importance, significant gaps remain regarding the application of e-health strategies to address sarcopenic obesity. This scoping review aims to map the current evidence on the use of e-health in addressing sarcopenic obesity in adults with overweight or obesity, identify barriers and facilitators to its implementation, and highlight areas for future research.

**Methods and analysis:**

The scoping review will be conducted in accordance with established methodological framework by the Joanna Briggs Institute (JBI), employing a comprehensive three-step search strategy across multiple databases and grey literature sources, including PubMed, Embase, Cochrane, CINAHL, Web of Science and Scopus. The inclusion criteria, framed by the Population-Concept-Context (PCC) framework, will focus on studies involving adults with sarcopenic obesity and interventions using e-health approaches in various healthcare contexts. A data extraction form will be used to guide the data extraction. Findings will be synthesised narratively and in tabular form, comprehensively mapping the current evidence and identifying key areas for future research.

**Ethics and dissemination:**

Ethical approval is not required as the review analyses publicly available data. Findings will be published in a peer-reviewed journal and presented at international conferences and scientific forums. The review will offer insights into e-health integration in sarcopenic obesity management, informing clinical practice, policy development and interdisciplinary collaboration.

**Study registration:**

This scoping review was registered with the Open Science Framework registry on 17 September 2024 (https://doi.org/10.17605/OSF.IO/9ND5A).

**STRENGTHS AND LIMITATIONS OF THIS STUDY**

- The review will follow the Joanna Briggs Institute methodology and Preferred Reporting Items for Systematic Review and Meta-Analysis-ScR guidelines to ensure methodological rigour.
- Inclusion criteria based on the PCC framework clearly define the population, concept and context for study selection.
- An experienced librarian will conduct the systematic literature search to ensure comprehensiveness and quality.
- In line with the scoping review guidance, a quality appraisal of the included studies will not be conducted.
- A limitation is that included studies will only be in English and Scandinavian languages, which may exclude relevant evidence.

## Introduction

 The public health burden of malnutrition is enormous. Globally, about 2.5 billion and 890 million adults have developed overweight or obesity, respectively.^1^ Overnutrition, leading to obesity, is associated with alterations in body composition and function. At the individual level, the double burden of malnutrition has continued to increase globally, typically marked by the co-existence of nutrition-related deficiencies and non-communicable diseases.^2^

Overweight and obesity are defined as abnormal or excess fat accumulation, which can negatively impact health.^1^ Despite different diagnostic criteria in existing literature, WHO’s definition of overweight and obesity as Body Mass Index (BMI) of ≥25 kg/m^2^ and ≥30 kg/m^2^, respectively, remains widely accepted.^3^

On the other hand, sarcopenia was first described by Rosenberg^4^ as a geriatric health condition characterised by loss of skeletal muscle mass and muscle function. The European Working Group on Sarcopenia in Older People (EWGSOP) recommendations highlight the presence of the following key factors indicative of varying degrees of sarcopenia**—**low muscle strength (grip strength <27 kg for men and <16 kg for women and chair stand >15 s for five rises for both sexes), low muscle quantity (appendicular skeletal muscle mass <20 kg for men and <15 kg for women) and poor physical performance (gait speed ≤0.8 m/s).^5^

Sarcopenic obesity, which manifests as body compositional changes showing a simultaneous increase in fat mass and decline in muscle mass/function, has become increasingly recognised. The interplay between obesity, sarcopenia and malnutrition—each with distinct health consequences—worsens when these conditions interact synergistically.^6^,^8^ This condition is associated with insulin resistance and increased risk of cardiovascular diseases,^9 10^ which exacerbates muscle loss in sarcopenia and thus further deteriorates the state.

Management of sarcopenic obesity focuses on improving the conditions associated with decreased muscle mass and elevated fat deposits.^11 12^ Dietary and lifestyle interventions remain the cornerstone of treatment; promoting physical activity and weight loss with optimal nutrition, particularly protein, fruits and vegetables, have been proven as effective strategies.^11 13 14^ Therefore, consistent and effective management of this condition can be strengthened by incorporating innovative techniques that facilitate care delivery. An example of such an innovation is e-health, a rapidly evolving approach that leverages digital and remote communication to enhance healthcare delivery.^15^

E-health refers to all forms of digital and remote communication in healthcare, entailing interactions that are not conducted in person, including services provided through the internet or telephone, mobile apps and other digital tools used to support healthcare delivery.^16^ E-health encompasses a new approach to delivering digital healthcare, facilitating increased patient engagement, accessibility and efficiency in healthcare. Previous systematic reviews have demonstrated that e-health can be a valuable tool in the management of several chronic diseases, such as diabetes mellitus,^17^ cardiovascular diseases and chronic lung diseases, partly through enabling education and increased engagement of patients, resulting in increased adherence to treatments and improved clinical outcomes.^18^

E-health holds significant potential in addressing obesity by improving healthcare accessibility, enhancing the quality and efficiency of patient-centred treatments and enabling long-term patient support.^19^ Early diagnosis of malnutrition can facilitate treatment and prevent many of its consequences, such as sarcopenic obesity.^20^ Due to its preventive and facilitated treatment options, significant benefits can be gained from developing e-health tools for managing malnutrition. Early detection and continuous monitoring of key health parameters, such as muscle mass, strength, physical activity levels and dietary intake, are enhanced through digitalisation.^21^ These digital tools help identify risks often overlooked due to a higher BMI, facilitating timely interventions tailored to the unique needs of individuals.

### Current knowledge gaps

Despite the aforementioned potential of e-health, significant knowledge gaps exist regarding its application in the management of sarcopenic obesity. This population remains underrepresented in research, and there is no consensus on best practices for integrating e-health into this context despite its growing use in various healthcare domains.^21^ A scoping review is therefore needed to map the current state of research and identify areas for further investigation. Most studies on malnutrition and sarcopenia have traditionally focused on undernourished or ageing populations without explicitly addressing adults with overweight or obesity.^21^,^23^ Sarcopenia in this demographic is frequently overlooked, commonly due to the obesity paradox**—**a misconception that obesity protects against muscle loss and nutritional deficiencies^24^**—**and the lack of knowledge about sarcopenic obesity and malnutrition pathophysiology among clinicians and researchers.^22^ Furthermore, even though there are specific criteria for the definition of sarcopenia, identifying the prevalence of sarcopenic obesity remains complex due to the lack of universally accepted diagnostic criteria.^22^ Consequently, these factors lead to the lack of tailored research examining how e-health can address these hidden conditions within this demographic.

Additionally, there is a dearth of research on the feasibility of integrating e-health tools into routine clinical workflows, particularly for individuals with overweight and obesity who may have different barriers to digital health adoption compared with other populations.^23^

A preliminary search of MEDLINE, the Cochrane Database of Systematic Reviews and Joanna Briggs Institute (JBI) Evidence Synthesis was conducted. No published systematic reviews, scoping reviews or protocols explicitly addressing this topic were identified at the time of the search.

### Aim of the scoping review

The objective of the scoping review is to map and identify the current evidence on the use of e-health in addressing sarcopenic obesity in adults with overweight or obesity.

## Research questions

1. What types and to what extent are e-health used for addressing sarcopenic obesity?
2. What impact does e-health have on addressing sarcopenic obesity?
3. What barriers and facilitators exist in using e-health to address sarcopenic obesity?

## Methods

The proposed scoping review will be conducted in accordance with the JBI methodology for scoping reviews.^25^,^27^ This review will be conducted in accordance with the Preferred Reporting Items for Systematic Review and Meta-Analysis (PRISMA) ScR checklist.^28^ The authors will systematically identify, review and map existing international evidence of relevance that used e-health in addressing sarcopenia in adults with overweight/obesity.

### Search strategy

When developing the study’s idea and concept, and before specifying the research question, initial limited searches of free text words were conducted in MEDLINE (PubMed).

A three-step search strategy will be employed in the review, as recommended by JBI.^25^ Initial and secondary searches will be conducted in close collaboration between the shared first author SG and an experienced university hospital librarian. The search strategy will aim to locate published and unpublished studies in English or Scandinavian languages (for feasibility reasons and to ensure accurate analysis). No date range is used in the search as we expect the inclusion of ‘e-health’ as a topic to naturally select relevant results. At the same time, we do not wish to exclude sources based on specific publication years, as this may result in the omission of relevant references. First, an initial limited search of MEDLINE (PubMed), Embase and CINAHL (EBSCO) for academic articles and recommended websites and guidelines for grey literature will be undertaken to identify relevant articles on the topic. The text words contained in the titles and abstracts of relevant articles and the index terms used to describe them will be used to develop a complete search strategy. The search strategy, including all identified keywords and index terms, will be adapted for each included database. In relation to unpublished and grey literature, a search will be conducted on Google, Google Scholar and various guidelines and recommendation websites. Lastly, the reference lists of all included reports and articles will be screened for additional evidence. Reviews that meet inclusion criteria will be included to examine the reference lists for relevant sources. The complete search strategy is provided in an online supplemental file.

### Inclusion criteria

The review question was formed according to the Population-Concept-Context (PCC) framework.

Population: This review will consider materials that include adults (age ≥18 years) with sarcopenic obesity or with data permitting classification as above, with mean BMI ≥25 kg/m²; if mean BMI <25 kg/m², inclusion requires clear evidence of excess adiposity by validated non-BMI measures (eg, body-fat %, waist circumference, fat mass index). Sarcopenic obesity manifests as body compositional changes showing a simultaneous increase in fat mass and a decline in muscle mass/function.

Concept: The scoping review includes materials on e-health as a professional approach for addressing sarcopenic obesity. E-health refers to all forms of digital and remote communication in healthcare, entailing interactions that are not conducted in person, including services, interventions, screening, monitoring, education, coaching or follow-up delivered exclusively or partly via digital/remote modalities (eg, telemedicine/video/phone, web portals, mobile apps/SMS, wearables/remote monitoring; synchronous or asynchronous).

Context: The scoping review includes material from where the individual with sarcopenic obesity is situated, that is, any healthcare setting (hospital, outpatient/primary care, community/long-term care). Both peer-reviewed and grey literature published in English or Scandinavian languages will be included.

### Exclusion criteria

Studies focusing on paediatric populations, pregnancy populations, as well as animal/in vitro studies. Also, cachexia, sarcopenia and populations with underweight without demonstrations of excess adiposity will be ineligible for inclusion. Interventions delivered exclusively in person (no substantive digital/remote component) or articles written in non-English/Scandinavian language will not be included.

### Operation definition of terms

The absence of a single, universally accepted diagnostic criterion for sarcopenic obesity will be mapped in the review. Following expert recommendations,^22 29 30^ studies are eligible if they include adults with both an adiposity component and a sarcopenia component, even when diagnostic cut-offs vary.^22^ We will include studies that either (a) explicitly label the condition ‘sarcopenic obesity’, or (b) define the population using established measures for both components:

Adiposity component: Mean BMI ≥25 kg/m² (ethnicity-specific thresholds accepted if stated), or validated indicators of excess adiposity (eg, high body-fat %, fat mass index, waist circumference) measured by dual-energy x-ray absorptiometry (DXA)/bioelectrical impedance analysis (BIA)/anthropometry.

Sarcopenia component: Low muscle strength (eg, handgrip or 5-chair stand), and/or low muscle quantity/quality (eg, appendicular skeletal muscle by DXA/BIA) and/or poor physical performance (eg, gait speed), consistent with major consensus frameworks (eg, EWGSOP).

For each included study, we will extract and present the criteria and cut-offs used for both components to enable comparison across definitions.

### Types of sources

The scoping review will consider materials such as research articles, methodological papers and clinical guidelines that report on sarcopenic obesity and e-health. Sources from any research methodology or grey literature will be considered in systematically searching, selecting and synthesising of knowledge.

### Source of evidence selection

Following the search, all identified citations will be collated and uploaded into EndNote 21,^31^ and duplicates will be removed. Following a pilot test, titles and abstracts will be screened against the inclusion criteria for the review. Then, the full text of the sources included will be assessed. Any disagreements between the reviewers at each stage of the selection process will be resolved through discussion until a consensus is reached. In case of significant disputes, an additional reviewer will be involved. The results of the search and the study inclusion process will be reported in full in the final scoping review and presented in a PRISMA flow diagram.^28^

### Data extraction

The Covidence extraction template 2.0 software will be used for data extraction, and a data extraction tool will be developed a priori by the reviewers, as per the JBI methodology.^25^,^27^ The data extraction tool will be provided. Key information will include specific details relevant to the review questions, including title, authors, year of publication, country of origin, aim, population and sample size, healthcare setting/context, methodology/design, the type and purpose of e-health in addressing sarcopenic obesity, findings related to professional interventions (eg, intervention type, content intensity, duration, monitoring, context of use), professional responsibilities, outcome as well as reported barriers and facilitators in the implementation of intervention. The extraction tool will be modified as necessary during a pilot process. All authors will independently extract data from a sample of the included evidence sources, and a cross-check will be conducted to ensure the consistency of the extracted data. Finally, the developed data extraction tool extracts data from all included sources. Any disagreements between the reviewers will be resolved through discussion or consensus with the assistance of an additional reviewer.

### Data analysis and presentation

Data from the extraction process will be mapped and presented in a tabular form divided into appropriate conceptual categories fitting the review questions (eg, intervention type, content, intensity and duration of intervention), including a basic numerical account of the amount, type and distribution of the evidence included in the review. Two independent researchers will conduct data analysis and discussions involving all authors. A narrative summary will accompany the tabulated results to describe how the findings relate to the research questions.

### Patient and public involvement

Patients and/or the public were not involved in the design, execution, reporting or dissemination of this research.

## Ethics and dissemination

This review involves analysis of already published and publicly available data. Therefore, ethical approval is not required, as the study will not involve human participants. The findings will be disseminated through publication in a peer-reviewed journal, such as BMJ, and presented at relevant international scientific conferences and other broadly accessible scientific forums. The review aims to provide critical insights into integrating e-health in the management of sarcopenic obesity, offering information to inform clinical practice, guide policy development and enhance patient care and interdisciplinary collaboration in contemporary healthcare systems.

### Study timeline

The review will be conducted over a period of 9 months, from 11 May 2025 to 27 February 2026.

## Supplementary material

10.1136/bmjopen-2025-103773 online supplemental file 1
