# Supplementary material for: Role of e-health in addressing sarcopenic obesity: a scoping review protocol
Source: BMJ Open. 2025 Nov 4;15(11):e103773. doi: 10.1136/bmjopen-2025-103773 (PMC12587994; doi:10.1136/bmjopen-2025-103773)
Supplement: online supplemental file 1 [file bmjopen-15-11-s001.docx]

Supplemental Material Files

We made a systematic literature search in the databases PubMed, Embase, Cochrane and CINAHL, Web of Science, and Scopus on December 6^th^, 2024. Table 1 presents an overview of the databases and results of the systematic search. Table 2 presents a PICO scheme of the search as conducted in PubMed. Tables 3-8 presents the search strategy in all databases, respectively.

Table 1. Databases and results

| **Database** | **Platform** | **Results** |
| --- | --- | --- |
| **PubMed** | PubMed.gov | 69 |
| **Embase** | Embase.com | 65 |
| **Cochrane** | Wiley | 45 |
| **CINAHL with Full Text** | EBSCOhost | 32 |
| **Web of Science** | Clarivate | 134 |
| **Scopus** | Scopus.com | 144 |
| **All**  **÷ duplicates with EndNote** |  | 489  259 |

Table 2. PICO scheme of search strategy in PubMed.

|  | **AND** | | |
| --- | --- | --- | --- |
| **OR** | Overweight [Mesh]  Overweight* [Title/Abstract]  Obese [Title/Abstract]  Obesit* [Title/Abstract]  **NOT**  Adolescent [Mesh]  Child [Mesh]  Infant [Mesh] | Sarcopenia [Mesh]  Sarcopeni* [Title/Abstract]  Osteosarcopeni* [Title/Abstract] | Telecommunications [Mesh]  Telecommunicat* [Title/Abstract]  Telehealth* [Title/Abstract]  Telemedicin* [Title/Abstract]  Digital* [Title/Abstract]  Ehealth* [Title/Abstract]  E-health* [Title/Abstract]  Mhealth* [Title/Abstract]  Mobile health* [Title/Abstract]  Technolog* [Title/Abstract]  Virtual* [Title/Abstract]  Video* [Title/Abstract]  Telephone* [Title/Abstract]  Smartphone* [Title/Abstract]  Tablet* [Title/Abstract]  SMS [Title/Abstract]  Text message [Title/Abstract]  App [Title/Abstract]  Apps [Title/Abstract]  Mobile application* [Title/Abstract] |

Table 3. Search strategy in PubMed.

| Search | Query | Results |
| --- | --- | --- |
| #4 | Search: (("Overweight"[Mesh] OR overweight*[Title/Abstract] OR obese[Title/Abstract] OR obesit*[Title/Abstract]) AND ("Sarcopenia"[Mesh] OR sarcopeni*[Title/Abstract] OR osteosarcopeni*[Title/Abstract])) AND ("Telecommunications"[Mesh] OR telecommunicat*[Title/Abstract] OR telehealth*[Title/Abstract] OR telemedicin*[Title/Abstract] OR digital*[Title/Abstract] OR ehealth*[Title/Abstract] OR e-health*[Title/Abstract] OR mhealth*[Title/Abstract] OR mobile health*[Title/Abstract] OR technolog*[Title/Abstract] OR virtual*[Title/Abstract] OR video*[Title/Abstract] OR telephone*[Title/Abstract] OR smartphone*[Title/Abstract] OR tablet*[Title/Abstract] OR sms[Title/Abstract] OR text message*[Title/Abstract] OR app[Title/Abstract] OR apps[Title/Abstract] OR mobile application*[Title/Abstract]) Sort by: Publication Date | [69](https://pubmed.ncbi.nlm.nih.gov/?term=%28%28%22Overweight%22%5BMesh%5D+OR+overweight%2A%5BTitle%2FAbstract%5D+OR+obese%5BTitle%2FAbstract%5D+OR+obesit%2A%5BTitle%2FAbstract%5D%29+AND+%28%22Sarcopenia%22%5BMesh%5D+OR+sarcopeni%2A%5BTitle%2FAbstract%5D+OR+osteosarcopeni%2A%5BTitle%2FAbstract%5D%29%29+AND+%28%22Telecommunications%22%5BMesh%5D+OR+telecommunicat%2A%5BTitle%2FAbstract%5D+OR+telehealth%2A%5BTitle%2FAbstract%5D+OR+telemedicin%2A%5BTitle%2FAbstract%5D+OR+digital%2A%5BTitle%2FAbstract%5D+OR+ehealth%2A%5BTitle%2FAbstract%5D+OR+e-health%2A%5BTitle%2FAbstract%5D+OR+mhealth%2A%5BTitle%2FAbstract%5D+OR+mobile+health%2A%5BTitle%2FAbstract%5D+OR+technolog%2A%5BTitle%2FAbstract%5D+OR+virtual%2A%5BTitle%2FAbstract%5D+OR+video%2A%5BTitle%2FAbstract%5D+OR+telephone%2A%5BTitle%2FAbstract%5D+OR+smartphone%2A%5BTitle%2FAbstract%5D+OR+tablet%2A%5BTitle%2FAbstract%5D+OR+sms%5BTitle%2FAbstract%5D+OR+text+message%2A%5BTitle%2FAbstract%5D+OR+app%5BTitle%2FAbstract%5D+OR+apps%5BTitle%2FAbstract%5D+OR+mobile+application%2A%5BTitle%2FAbstract%5D%29&sort=pubdate&size=200&ac=no) |
| #3 | Search: "Telecommunications"[Mesh] OR telecommunicat*[Title/Abstract] OR telehealth*[Title/Abstract] OR telemedicin*[Title/Abstract] OR digital*[Title/Abstract] OR ehealth*[Title/Abstract] OR e-health*[Title/Abstract] OR mhealth*[Title/Abstract] OR mobile health*[Title/Abstract] OR technolog*[Title/Abstract] OR virtual*[Title/Abstract] OR video*[Title/Abstract] OR telephone*[Title/Abstract] OR smartphone*[Title/Abstract] OR tablet*[Title/Abstract] OR sms[Title/Abstract] OR text message*[Title/Abstract] OR app[Title/Abstract] OR apps[Title/Abstract] OR mobile application*[Title/Abstract] Sort by: Publication Date | [1,573,718](https://pubmed.ncbi.nlm.nih.gov/?term=%22Telecommunications%22%5BMesh%5D+OR+telecommunicat%2A%5BTitle%2FAbstract%5D+OR+telehealth%2A%5BTitle%2FAbstract%5D+OR+telemedicin%2A%5BTitle%2FAbstract%5D+OR+digital%2A%5BTitle%2FAbstract%5D+OR+ehealth%2A%5BTitle%2FAbstract%5D+OR+e-health%2A%5BTitle%2FAbstract%5D+OR+mhealth%2A%5BTitle%2FAbstract%5D+OR+mobile+health%2A%5BTitle%2FAbstract%5D+OR+technolog%2A%5BTitle%2FAbstract%5D+OR+virtual%2A%5BTitle%2FAbstract%5D+OR+video%2A%5BTitle%2FAbstract%5D+OR+telephone%2A%5BTitle%2FAbstract%5D+OR+smartphone%2A%5BTitle%2FAbstract%5D+OR+tablet%2A%5BTitle%2FAbstract%5D+OR+sms%5BTitle%2FAbstract%5D+OR+text+message%2A%5BTitle%2FAbstract%5D+OR+app%5BTitle%2FAbstract%5D+OR+apps%5BTitle%2FAbstract%5D+OR+mobile+application%2A%5BTitle%2FAbstract%5D&sort=pubdate&size=200&ac=no) |
| #2 | Search: "Sarcopenia"[Mesh] OR sarcopeni*[Title/Abstract] OR osteosarcopeni*[Title/Abstract] Sort by: Publication Date | [21,902](https://pubmed.ncbi.nlm.nih.gov/?term=%22Sarcopenia%22%5BMesh%5D+OR+sarcopeni%2A%5BTitle%2FAbstract%5D+OR+osteosarcopeni%2A%5BTitle%2FAbstract%5D&sort=pubdate&size=200&ac=no) |
| #1 | Search: "Overweight"[Mesh] OR overweight*[Title/Abstract] OR obese[Title/Abstract] OR obesit*[Title/Abstract] Sort by: Publication Date | [494,270](https://pubmed.ncbi.nlm.nih.gov/?term=%22Overweight%22%5BMesh%5D+OR+overweight%2A%5BTitle%2FAbstract%5D+OR+obese%5BTitle%2FAbstract%5D+OR+obesit%2A%5BTitle%2FAbstract%5D&sort=pubdate&size=200&ac=no) |

Table 4. Search strategy in Embase.

| No. | Query | Results |
| --- | --- | --- |
| #5 | #4 NOT ('conference abstract'/it OR 'conference paper'/it OR 'conference review'/it) | 65 |
| #4 | #1 AND #2 AND #3 | 123 |
| #3 | 'mass communication'/exp/mj OR telecommunicat*:ti,ab,kw OR telehealth*:ti,ab,kw OR telecare:ti,ab,kw OR telemedicin*:ti,ab,kw OR digital*:ti,ab,kw OR ehealth*:ti,ab,kw OR 'e-health*':ti,ab,kw OR mhealth*:ti,ab,kw OR 'mobile health*':ti,ab,kw OR technolog*:ti,ab,kw OR virtual*:ti,ab,kw OR video*:ti,ab,kw OR telephone*:ti,ab,kw OR smartphone*:ti,ab,kw OR tablet*:ti,ab,kw OR sms:ti,ab,kw OR 'text message*':ti,ab,kw OR app:ti,ab,kw OR apps:ti,ab,kw OR 'mobile application*':ti,ab,kw | 2146195 |
| #2 | 'sarcopenia'/de OR sarcopeni*:ti,ab,kw OR osteosarcopeni*:ti,ab,kw | 34594 |
| #1 | 'obesity'/exp/mj OR overweight*:ti,ab,kw OR obese:ti,ab,kw OR obesit*:ti,ab,kw | 716889 |

Table 5. Search strategy in Cochrane.

| ID | Search | Hits |
| --- | --- | --- |
| #1 | MeSH descriptor: [Overweight] explode all trees | 25843 |
| #2 | (overweight* OR obese OR obesity):ti,ab,kw | 63534 |
| #3 | #1 OR #2 | 63639 |
| #4 | MeSH descriptor: [Sarcopenia] explode all trees | 986 |
| #5 | (sarcopeni* OR osteosarcopeni*):ti,ab,kw | 2907 |
| #6 | #4 OR #5 | 2907 |
| #7 | MeSH descriptor: [Telecommunications] explode all trees | 12090 |
| #8 | (telecommunicat* OR telehealth* OR telecare OR telemedicin* OR digital* OR ehealth* OR e-health* OR mhealth* OR (mobile NEXT health*) OR technolog* OR virtual* OR video* OR telephone* OR smartphone* OR tablet* OR sms OR (text NEXT message*) OR app OR apps OR (mobile NEXT application*)):ti,ab,kw | 203772 |
| #9 | #7 OR #8 | 205213 |
| #10 | #3 AND #6 AND #9 | 45 |

Table 6. Search strategy in CINAHL.

| # | Query | Results |
| --- | --- | --- |
| S4 | S1 AND S2 AND S3 | 32 |
| S3 | (MH "Telecommunications+") OR ( telecommunicat* OR telehealth* OR telecare OR telemedicin* OR digital* OR ehealth* OR e-health* OR mhealth* OR mobile health* OR technolog* OR virtual* OR video* OR telephone* OR smartphone* OR tablet* OR sms OR text message* OR app OR apps OR mobile application* ) | 563,987 |
| S2 | (MH "Sarcopenia") OR ( sarcopeni* OR osteosarcopeni* ) | 7,850 |
| S1 | (MH "Obesity+") OR ( overweight* OR obese OR obesit* ) | 169,819 |

Table 7. Search strategy in Web of Science.

| # | Query | Hits |
| --- | --- | --- |
| 1 | overweight* OR obese OR obesit* (Topic) and sarcopeni* OR osteosarcopeni* (Topic) and telecommunicat* OR telehealth* OR telecare OR telemedicin* OR digital* OR ehealth* OR "e-health*" OR mhealth* OR "mobile health*" OR technolog* OR virtual* OR video* OR telephone* OR smartphone* OR tablet* OR sms OR "text message*" OR app OR apps OR "mobile application*" (Topic) | [134](https://www-webofscience-com.auh.aub.aau.dk/wos/woscc/summary/5203af4b-caed-43d6-8362-d8205ba5f02f-0133084ae9/relevance/1) |

Table 8. Search strategy in Scopus.

| # | Query | Hits |
| --- | --- | --- |
| 2 | ( TITLE-ABS-KEY ( overweight* OR obese OR obesit* ) AND TITLE-ABS-KEY ( sarcopeni* OR osteosarcopeni* ) AND TITLE-ABS-KEY ( telecommunicat* OR telehealth* OR telecare OR telemedicin* OR digital* OR ehealth* OR "e-health*" OR mhealth* OR "mobile health*" OR technolog* OR virtual* OR video* OR telephone* OR smartphone* OR tablet* OR sms OR "text message*" OR app OR apps OR "mobile application*" ) ) AND ( LIMIT-TO ( SRCTYPE , "j" ) ) | 144 |
| 1 | ( TITLE-ABS-KEY ( overweight* OR obese OR obesit* ) AND TITLE-ABS-KEY ( sarcopeni* OR osteosarcopeni* ) AND TITLE-ABS-KEY ( telecommunicat* OR telehealth* OR telecare OR telemedicin* OR digital* OR ehealth* OR "e-health*" OR mhealth* OR "mobile health*" OR technolog* OR virtual* OR video* OR telephone* OR smartphone* OR tablet* OR sms OR "text message*" OR app OR apps OR "mobile application*" ) ) | 148 |
