# Supplementary material for: Role of e-health in addressing sarcopenic obesity: a scoping review protocol
Source: BMJ Open. 2025 Nov 4;15(11):e103773. doi: 10.1136/bmjopen-2025-103773 (PMC12587994; doi:10.1136/bmjopen-2025-103773)
Supplement: Supplementary file 2 [file bmjopen-2025-103773.reviewer_comments.pdf]

PEER REVIEW HISTORY

BMJ Open publishes all reviews undertaken for accepted manuscripts. Reviewers are asked to complete a checklist review form and are provided with free text boxes to elaborate on their assessment. These free text comments are reproduced below.

ARTICLE DETAILS

Title (Provisional)

The role of e-health in addressing sarcopenic obesity-a scoping review protocol

Authors

Nørtoft, Mette; Graungaard, Signe; Iheme, Gideon; Karagianni, Vasiliki; Bereczky, Rebeka; Ellegaard, Lars; Boström, Anne-Marie

---

VERSION 1 - REVIEW

**Reviewer**	**1**
**Name**	**Li, Zengning**
**Affiliation**	**Hebei Key Laboratory of Nutrition and Health**
**Date**	**16-Sep-2025**
**COI**	

The article focuses on individuals with sarcopenic obesity and systematically reviews the role of e-health in the health management of this population. It holds certain clinical significance, and the basic structure of the review protocol is clear and well-organized. However, the following issues need further clarification:

1.Methods

（1） What is the definition/criterion for sarcopenic obesity? During Literature screening, was this definition taken into account?

（2） In Appendix Table 2, the PubMed search strategy contains “NOT Adult”, which conflicts with the Population definition described in the text. Please verify whether the search string is correct.

（3） It is recommended to further clarify the inclusion and exclusion criteria for the literature.

2.Typographical error

In the appendix, Table 1 is incorrectly labeled as “Table 2”. Please carefully check the manuscript to avoid such errors.

VERSION 1 - AUTHOR RESPONSE

Reviewer´s comments

Dear Reviewer, thank you for the commitment and dedication to improve the quality of our work, we have revised the study following your valuable comments, find attached our response below.

1.Methods

- （1）What is the definition/criterion for sarcopenic obesity? During Literature screening, was this definition taken into account?

Response: Thank you for your question. We have provided clarity to the definition(s) of sarcopenic obesity that informed the screening process. See page 7.

2）In Appendix Table 2, the PubMed search strategy contains “NOT Adult”, which conflicts with the Population definition described in the text. Please verify whether the search string is correct.

Response: Thank you for this correction. This is an error that has been removed, as adult were part of the study´s inclusion criteria. See page 15.

（3）It is recommended to further clarify the inclusion and exclusion criteria for the literature.

Response: Thank you for pointing at this. All authors had already some discussion about clarifying more and being more specific. We have now specified in the text, please see pages 6 and 7.

2.Typographical error

In the appendix, Table 1 is incorrectly labeled as “Table 2”. Please carefully check the manuscript to avoid such errors.

Response: Thank you for this correction, changes are made. See page 14.

If you have selected ‘Yes’ above, please provide details of any competing interests.: Not applicable

Response: We have already provided comments on the absence of competing interest, this has been revised further, see page 10. We will make sure to select “No” during the resubmission process.
